# Superfluid density, Josephson relation and pairing fluctuations in a multi-component fermion superfluid

**DOI:** 10.1038/s41598-021-01261-y

**Published:** 2021-11-08

**Authors:** Yi-Cai Zhang

**Affiliations:** School of Physics and Materials Science, Guangzhou University, Guangzhou, 510006 People’s Republic of China

**Keywords:** Atomic and molecular physics, Condensed-matter physics, Quantum physics

## Abstract

In this work, a Josephson relation is generalized to a multi-component fermion superfluid. Superfluid density is expressed through a two-particle Green function for pairing states. When the system has only one gapless collective excitation mode, the Josephson relation is simplified, which is given in terms of the superfluid order parameters and the trace of two-particle normal Green function. In addition, it is found that the matrix elements of two-particle Green function is directly related to the matrix elements of the pairing fluctuations of superfluid order parameters. Furthermore, in the presence of inversion symmetry, the superfluid density is given in terms of the pairing fluctuation matrix. The results of the superfluid density in Haldane model show that the generalized Josephson relation can be also applied to a multi-band fermion superfluid in lattice.

## Introduction

The superfluid density $\rho _s$ and order parameter $n_0$ in superfluid liquid Helium-4 are two closely related^1,2^, but different concepts^3,4^. However, they can connect with each other through a Josephson relation^5–7^, i.e.,

1 $$\begin{aligned} \rho _s=-\mathrm{lim}_{{\hbox {q}\rightarrow 0}}\frac{n_0m}{q^2 G(\mathbf{q} ,0)}, \end{aligned}$$

where $\rho _s$ is superfluid density (particle number per unit volume), $n_0$ is order parameter (condensate density) in liquid Helium-4. $G(\mathbf{q} ,0)$ is normal single-particle Green function at zero frequency, *m* is atomic mass of Helium-4. The above equation indicates that the Green function diverges as wave vector $q\rightarrow 0$. Such a divergence of $1/q^2$ in Green function is quite a universal phenomenon which can occur in many systems, e.g., superfluid Helium-4, superconductor and ferromagnet^8^. The above Josephson relation in superfluid system can be viewed as a manifestation of Bogoliubov’s “$1/q^2$” theorem in spontaneously symmetry breaking system^7^. It also has close connections with the absence of long ranged order (e.g., condensation) at finite temperature in one and two dimensions^9,10^.

Its possible generalization in two-component fermion superfluid has been firstly investigated by Taylor^11^. Using auxiliary-field approach, Dawson et al. also derived a Josephson relation

2 $$\begin{aligned} \rho _s=-\mathrm{lim}_{{\hbox {q}\rightarrow 0}}\frac{4 \Delta ^{2}m}{q^2 G_{II}(\mathbf{q} ,0)}, \end{aligned}$$

which holds for both bosonic and fermion superfluids^12^. Here $\Delta$ is superfluid order parameter (pairing gap in superconductor) and $G_{II}(\mathbf{q} ,\omega )$ is two-particle Green function for pairing states in conventional two-component fermion superfluid. It is found that the superfluid density is determined by superfluid order parameter and the behaviors of two-particle Green function at long wave length limit. In comparison with bosonic superfluid, the above formula shows that the two-particle Green function replace the corresponding single-particle Green function of bosonic superfluid. In addition, pairing gap $\Delta$ plays the roles of order parameter in fermion superfluid.

The superfluid properties in multi-component (or multi-band) fermion system have attracted a great interests^13–21^. The exotic pairing mechanism in Fermi gas with SU(N) invariant interaction has been proposed^22–29^, dependent on interaction and chemical potential, which can show coexistence of superfluidity and magnetism^30^. Another interesting example of multi-band fermion system is twisted bilayer graphene^31,32^. It is shown that, there exists superconductivity^33^ in this system. Its superfluid weight (which is superfluid density up to a constant) have been investigated intensively^34–38^. Inspired by above studies, in this paper we investigate the superfluid density for multi-component fermion superfluid. A generalized formula of Josephson relation for multi-component bosons has been given in Ref.^39^. A natural question arises: does there exist a similar relation for a multi-component fermion superfluid or superconductor?

In this paper, we give a generalized Josephson relation for a multi-component fermion superfluid system. It is found that we can take a similar method as done in bosonic system to get the general results for fermions. To be specific, the superfluid density can be expressed in terms of two-particle Green functions. Furthermore, when there is only one gapless collective mode, the superfluid density is determined by the superfluid order parameter and the trace of two-particle Green function. In addition, in the presence of spatial inversion symmetry, the two-particle Green function is directly related to fluctuation matrix of order parameters. Furthermore, it is found that the generalized Josephson relation can be also applied to a multi-band lattice system.

### Results

#### Josephson relation in conventional two component fermion superfluid

First of all, we consider two (spin) component fermion superfluid system with short-ranged attractive interactions. The Hamiltonian is

3 $$\begin{aligned}&H=H_0+V_{int},\nonumber \\&H_0=\int d^3\mathbf{r} \sum _{\sigma =\uparrow ,\downarrow }\psi ^{\dag }_{\sigma }(\mathbf{r} )[-\frac{\hbar ^2\nabla ^2}{2m}-\mu ]\psi _{\sigma }(\mathbf{r} ),\nonumber \\&V_{int}=\frac{g}{V}\int d^3\mathbf{r} \psi ^{\dag }_{\uparrow }(\mathbf{r} )\psi ^{\dag }_{\downarrow }(\mathbf{r} )\psi _{\downarrow }(\mathbf{r} )\psi _{\uparrow }(\mathbf{r} ), \end{aligned}$$

where *m* is particle mass, $\mu$ is chemical potential, *V* is volume of system and *g* is interaction strength parameter between two different (spin) components. In the rest of paper, we set $\hbar =V=1$ for simplifications.

Similarly as bosonic superfluid^39^, here we outline how to get the above relation Eq. (2) in the two-component fermion system. First of all, it is well known that the superfluid order parameter (or pairing gap in superconductor) in two-component fermions is

4 $$\begin{aligned} \Delta (\mathbf{r} )\equiv \langle {\hat{\Delta }}(\mathbf{r} )\rangle =\Delta , \end{aligned}$$

where ${\hat{\Delta }}(\mathbf{r} )=g\psi _{\downarrow }(\mathbf{r} )\psi _{\uparrow }(\mathbf{r} )$ is operator of order parameter.

In addition, when the superfluid order parameter $\Delta$ undergoes a small phase twist $e^{2i \delta \theta (\mathbf{r} )}$^11,40^, the variation (fluctuation) of superfluid order parameter is

5 $$\begin{aligned}&\Delta (\mathbf{r} )\rightarrow \Delta (\mathbf{r} ) e^{2i\delta \theta (\mathbf{r} )},\nonumber \\&\delta \Delta (\mathbf{r} )=\Delta (\mathbf{r} )e^{2i\delta \theta (\mathbf{r} )}-\Delta (\mathbf{r} )\simeq 2i\Delta \delta \theta (\mathbf{r} ), \end{aligned}$$

where $\delta \theta (\mathbf{r} )$ is a real function which denotes the small phase twist.

On the other hand, a phase gradient of superfluid order parameter would induce a superfluid current (superflow)^41^, namely

6 $$\begin{aligned} \delta \mathbf{j} (\mathbf{r} )\equiv \rho _{s}\mathbf {\nabla }\delta \theta (\mathbf{r} )/m=\rho _{s}{} \mathbf{v} _s, \end{aligned}$$

where we define superfluid velocity as the gradient of phase, i.e.,$\mathbf{v} _s\equiv \mathbf {\nabla } \delta \theta (\mathbf{r} )/m$ and superfluid density $\rho _s$ as the coefficient before $\mathbf{v} _s$ in the current $\delta \mathbf{j} (\mathbf{r} )$. We will see the connection between the above two equations (Eqs. (6), (5)) would result in the Josephson relation.

In order to get the relationship between superfluid density $\rho _s$ and order parameter $\Delta$, similarly as bosonic case^9,39,42^, here we need a perturbation which include operator of order parameter ${\hat{\Delta }}(\mathbf{r} )=g\psi _{\downarrow }(\mathbf{r} )\psi _{\uparrow }(\mathbf{r} )$ and its adjoint ${\hat{\Delta }}^{\dag }(\mathbf{r} )=g\psi ^{\dag }_{\uparrow }(\mathbf{r} )\psi ^{\dag }_{\downarrow }(\mathbf{r} )$, i.e.,

7 $$\begin{aligned} H' & = \!\!\int \!\! d^3\mathbf{r }[e^{i(\mathbf{q} \cdot \mathbf{r }-\omega t)+\epsilon t}\xi {\hat{\Delta }}^{\dag }(\mathbf{r} )+e^{-i(\mathbf{q} \cdot \mathbf{r} -\omega t)+\epsilon t}\xi ^* {\hat{\Delta }}(\mathbf{r} )], \nonumber \\ & =\xi {\hat{\Delta }}^{\dag }_\mathbf{q }e^{-i\omega t+\epsilon t}+\xi ^*{\hat{\Delta }}_\mathbf{q }e^{i\omega t+\epsilon t}, \end{aligned}$$

where $\xi$ is a small complex number, ${\hat{\Delta }}_\mathbf{q }=\sum _{k}g\psi _{\downarrow \mathbf{q}+k }\psi _{\uparrow \mathbf -k }$ is fluctuation operator of order parameter in momentum space and we use relation $\psi _\sigma (\mathbf{r} )=\sum _\mathbf{k} \psi _{\sigma \mathbf{k} }e^{i\mathbf{k} \cdot \mathbf{r} }$. Here we add an infinitesimal positive number $\epsilon \rightarrow 0_+$ in the above exponential which corresponds to choose a boundary condition that the perturbation is very slowly added to the system^43^.

We assume initially the system is in the ground state $|0\rangle$, and then slowly turn on perturbation $H'$, the wave function can be written as

8 $$\begin{aligned}&|\Psi (t)\rangle =\sum _n a_n(t)e^{-iE_nt}|n\rangle , \end{aligned}$$

where $a_n(t\rightarrow -\infty )=\delta _{n,0}$ and $H|n\rangle =E_n|n\rangle$, *H* is unperturbed Hamiltonian. $|n\rangle$ and $E_n$ are eigenstate and eigenenergy respectively. Using perturbation theory, we get

9 $$\begin{aligned}&|\Psi (t)\rangle \simeq |0\rangle e^{-iE_0 t}+\sum _{n {\ne} 0} a_n(t)e^{-iE_nt}|n\rangle ,\nonumber \\&a_n(t)=\frac{1}{i}\int _{-\infty }^{t} d\tau H^{\prime}_{n0}(\tau )e^{i\omega _{n0}\tau }\nonumber \\&\!\!\quad \quad \;\;=\!\!\left[ \frac{\xi \langle n|{\hat{\Delta }}^{\dag }_\mathbf{q }|0\rangle e^{-i(\omega +i\epsilon -\omega _{n0})t}}{\omega +i\epsilon -\omega _{n0}}-\frac{\xi ^*\langle n|{\hat{\Delta }}_\mathbf{q }|0\rangle e^{i(\omega -i\epsilon +\omega _{n0})t}}{\omega -i\epsilon +\omega _{n0}}\right] , \end{aligned}$$

where $\omega _{n0}=E_n-E_0$. The changes of order parameter $\langle \psi _{\downarrow }\psi _{\uparrow }(\mathbf{r} )\rangle$ and current $\mathbf{j} (\mathbf{r} )$ are respectively

10 $$\begin{aligned}&\delta \Delta (\mathbf{r} )=\delta \langle {\hat{\Delta }}(\mathbf{r} )\rangle \nonumber \\&\!\!\qquad \quad =\,\!\!\xi e^{-i(\omega +i\epsilon )t}[\frac{\langle 0|{\hat{\Delta }}(\mathbf{r} )|n\rangle \langle n|{\hat{\Delta }}^{\dag }_\mathbf{q }|0\rangle }{\omega +i\epsilon -\omega _{n0}}\!\!-\!\!\frac{\langle 0|{\hat{\Delta }}^{\dag }_\mathbf{q }|n\rangle \langle n|{\hat{\Delta }}(\mathbf{r} )|0\rangle }{\omega +i\epsilon +\omega _{n0}}]\nonumber \\&\qquad \qquad \!\!+\,\!\!\xi ^* e^{i(\omega -i\epsilon )t}[\frac{\langle 0|{\hat{\Delta }}_\mathbf{q }|n\rangle \langle n|{\hat{\Delta }}(\mathbf{r} )|0\rangle }{\omega -i\epsilon -\omega _{n0}}\!\!-\!\!\frac{\langle 0|{\hat{\Delta }}(\mathbf{r} )|n\rangle \langle n|{\hat{\Delta }}_\mathbf{q }|0\rangle }{\omega -i\epsilon +\omega _{n0}}],\nonumber \\&\delta \mathbf{j} (\mathbf{r} )=\xi e^{-i(\omega +i\epsilon )t}[\frac{\langle 0|\mathbf{j} (\mathbf{r} )|n\rangle \langle n|{\hat{\Delta }}^{\dag }_\mathbf{q }|0\rangle }{\omega +i\epsilon -\omega _{n0}}-\frac{\langle 0|{\hat{\Delta }}^{\dag }_\mathbf{q }|n\rangle \langle n|\mathbf{j} (\mathbf{r} )|0\rangle }{\omega +i\epsilon +\omega _{n0}}]\nonumber \\&\qquad \quad +\,\xi ^* e^{i(\omega -i\epsilon )t}[\frac{\langle 0|{\hat{\Delta }}_\mathbf{q }|n\rangle \langle n|\mathbf{j} (\mathbf{r} )|0\rangle }{\omega -i\epsilon -\omega _{n0}}-\frac{\langle 0|\mathbf{j} (\mathbf{r} )|n\rangle \langle n|{\hat{\Delta }}_\mathbf{q }|0\rangle }{\omega -i\epsilon +\omega _{n0}}]. \end{aligned}$$

In the following, we assume the system has translational invariance and total momentum is a good quantum number. So state $|n\rangle$ is also eigenstate of total momentum $\mathbf{P} =\sum _{\sigma \mathbf{k} } \mathbf{k} \psi _{\sigma \mathbf{k} }^{\dag }\psi _{\sigma \mathbf{k} }$, e.g., $\mathbf{P} |n\rangle =\mathbf{q} _n|n\rangle$ with eigenvalue $\mathbf{q} _n$. On other hand, from commutation relations

$$\begin{aligned}&{[}{} \mathbf{P} ,{\hat{\Delta }}_\mathbf{q }^{\dag }]|n\rangle =\{\mathbf{P }{\hat{\Delta }}_\mathbf{q }^{\dag }-{\hat{\Delta }}_\mathbf{q }^{\dag }{} \mathbf{P} \}|n\rangle =\mathbf{q }{\hat{\Delta }}_\mathbf{q }^{\dag }|n\rangle ,\\&{[}{} \mathbf{P} ,{\hat{\Delta }}_\mathbf{q }]|n\rangle =\{\mathbf{P }{\hat{\Delta }}_\mathbf{q }-{\hat{\Delta }}_\mathbf{q }{} \mathbf{P} \}|n\rangle =-\mathbf{q} {\hat{\Delta }}_\mathbf{q }|n\rangle , \end{aligned}$$

we see ${\hat{\Delta }}_\mathbf{q }^{\dag }|n\rangle$ and ${\hat{\Delta }}_\mathbf{q }|n\rangle$ are also eigenstates of $\mathbf{P}$, with momenta $\mathbf{q} _n+\mathbf{q}$ and $\mathbf{q} _n-\mathbf{q}$, respectively. Using relations ${\hat{\Delta }}(\mathbf{r} )=\sum _\mathbf{q} {\hat{\Delta }}_\mathbf{q} e^{i\mathbf{q} \cdot \mathbf{r} }$, $\langle 0|{\hat{\Delta }}_\mathbf{q '}|n\rangle \langle n|{\hat{\Delta }}_\mathbf{q }^{\dag }|0\rangle =\delta _\mathbf{q ,\mathbf{q} '}|\langle 0|{\hat{\Delta }}_\mathbf{q} |n\rangle |^2$, $\langle 0|{\hat{\Delta }}_\mathbf{q }^{\dag }|n\rangle \langle n|{\hat{\Delta }}_\mathbf{q '}|0\rangle =\delta _\mathbf{q ,\mathbf{q} '}|\langle 0|{\hat{\Delta }}_\mathbf{q }^{\dag }|n\rangle |^2$, $\langle 0|{\hat{\Delta }}_\mathbf{q }|n\rangle \langle n|{\hat{\Delta }}_\mathbf{q '}|0\rangle =\delta _\mathbf{q ,-\mathbf{q} '}\langle 0|{\hat{\Delta }}_\mathbf{q }|n\rangle \langle n|{\hat{\Delta }}_{-\mathbf{q} }|0\rangle$ and $\langle 0|{\hat{\Delta }}_\mathbf{q '}|n\rangle \langle n|{\hat{\Delta }}_\mathbf{q }|0\rangle =\delta _\mathbf{q ,-\mathbf{q} '}\langle 0|{\hat{\Delta }}_{-\mathbf{q} }|n\rangle \langle n|{\hat{\Delta }}_\mathbf{q }|0\rangle$, the change of order parameter can be written as

11 $$\begin{aligned} \delta \Delta (\mathbf{r} )&=\xi e^{i\mathbf{q} \cdot \mathbf{r} -i(\omega +i\epsilon )t} G_{II}(\mathbf{q} ,\omega +i\epsilon )\nonumber \\&\quad +\xi ^* e^{-i\mathbf{q} \cdot \mathbf{r} +i(\omega -i\epsilon )t}F_{II}(\mathbf{q} ,\omega -i\epsilon ), \end{aligned}$$

where

12 $$\begin{aligned}&G_{II}(\mathbf{q} ,\omega +i\epsilon )=\sum _n[\frac{|\langle 0|{\hat{\Delta }}_\mathbf{q} |n\rangle |^2}{\omega +i\epsilon -\omega _{n0}} -\frac{|\langle 0|{\hat{\Delta }}^{\dag }_\mathbf{q} |n\rangle |^2}{\omega +i\epsilon +\omega _{n0}}],\nonumber \\&F_{II}(\mathbf{q} ,\omega -i\epsilon )\nonumber \\&\quad =\sum _n\left[ \frac{\langle 0|{\hat{\Delta }}_\mathbf{q }|n\rangle \langle n|{\hat{\Delta }}_{-\mathbf{q} }|0\rangle }{\omega -i\epsilon -\omega _{n0}}-\frac{\langle 0|{\hat{\Delta }}_{-\mathbf{q} }|n\rangle \langle n|{\hat{\Delta }}_\mathbf{q }|0\rangle }{\omega -i\epsilon +\omega _{n0}}\right] , \end{aligned}$$

is two-particle normal (anomalous) Green function for pairing states^44,45^.

Taking zero-frequency ($\omega \pm i \epsilon =0$) limit,

13 $$\begin{aligned}&\delta \Delta (\mathbf{r} )=\xi e^{i\mathbf{q} \cdot \mathbf{r} } G_{II}(\mathbf{q} ,0)+\xi ^* e^{-i\mathbf{q} \cdot \mathbf{r} }F_{II}(\mathbf{q} ,0). \end{aligned}$$

For two-component neutral fermions, the order parameter $\Delta (\mathbf{r} )=g\langle \psi _{\downarrow }(\mathbf{r} )\psi _{\uparrow }(\mathbf{r} )\rangle =\Delta$ can be taken as a real number, and the low energy collective excitation is Anderson–Bogoliubov phonon. Similarly as bosonic case^39^, it can be shown that $F_{II}(\mathbf{q} ,0)=-G_{II}(\mathbf{q} ,0)$ as $q\rightarrow 0$ (see “Discussions” section), so finally

14 $$\begin{aligned} \delta \Delta (\mathbf{r} )&=G_{II}(\mathbf{q} ,0)[\xi e^{i\mathbf{q} \cdot \mathbf{r} } -\xi ^* e^{-i\mathbf{q} \cdot \mathbf{r} }],\nonumber \\&=2i\alpha G_{II}(\mathbf{q} ,0)sin(\mathbf{q} \cdot \mathbf{r} +\phi ), \end{aligned}$$

where we set $\xi \equiv \alpha e^{i\phi }$ with amplitude $\alpha$ and phase $\phi$.

Similarly using commutation relation

15 $$\begin{aligned}&[\mathbf{P} _i,\mathbf{j} _\mathbf{q j}]=-\mathbf{q} _i \mathbf{j} _\mathbf{q j}, \end{aligned}$$

where indices $i,j=x,y,z$, current fluctuation operator $\mathbf{j} _\mathbf{q }=\sum _{\sigma \mathbf{k} } [(\mathbf{k} +\mathbf{q} /2)]/m\psi ^{\dag }_{\sigma \mathbf{k} }\psi _{\sigma \mathbf{k} +\mathbf{q} }$, and the translational invariance, we conclude $\mathbf{j} _\mathbf{q }|n\rangle$ is also eigenstate of $\mathbf{P}$, with momentum $\mathbf{q} _n-\mathbf{q}$. Using the fact of $\mathbf{j} (\mathbf{r} )=\sum _\mathbf{q} \mathbf{j} _\mathbf{q} e^{i\mathbf{q} \cdot \mathbf{r} }$, $\langle 0|\mathbf{j} _\mathbf{q '}|n\rangle \langle n|{\hat{\Delta }}_\mathbf{q }^{\dag }|0\rangle =\delta _\mathbf{q ,\mathbf{q} '}\langle 0|\mathbf{j} _\mathbf{q} |n\rangle \langle n|{\hat{\Delta }}_\mathbf{q }^{\dag }|0\rangle$ and $\langle 0|{\hat{\Delta }}_\mathbf{q }^{\dag }|n\rangle \langle n|\mathbf{j} _\mathbf{q '}|0\rangle =\delta _\mathbf{q ,\mathbf{q} '}\langle 0|{\hat{\Delta }}_\mathbf{q }^{\dag }|n\rangle \langle n|\mathbf{j} _\mathbf{q }|0\rangle$, the current is

16 $$\begin{aligned} \delta \mathbf{j} (\mathbf{r} )=\xi e^{i\mathbf{q} \cdot \mathbf{r} -i(\omega +i\epsilon )t}{} \mathbf{B} (\mathbf{q} ,\omega +i\epsilon )+h.c., \end{aligned}$$

where *h*.*c*. denotes Hermitian (complex) conjugate and

17 $$\begin{aligned} \mathbf{B} (\mathbf{q} ,\omega +i\epsilon )\equiv \sum _n\left[ \frac{\langle 0|\mathbf{j} _q|n\rangle \langle n|\Delta ^{\dag }_\mathbf{q }|0\rangle }{\omega +i\epsilon -\omega _{n0}}-\frac{\langle 0|\Delta ^{\dag }_\mathbf{q} |n\rangle \langle n|\mathbf{j} _\mathbf{q} |0\rangle }{\omega +i\epsilon +\omega _{n0}}\right] . \end{aligned}$$

When $$\omega \pm i\epsilon =0$$,

18 $$\begin{aligned} \delta \mathbf{j} (\mathbf{r} )=[\xi e^{i\mathbf{q} \cdot \mathbf{r} }{} \mathbf{B} (\mathbf{q} ,0) +h.c.]. \end{aligned}$$

Using continuity equation $\frac{\partial \rho (\mathbf{r} ,t)}{\partial t}+\mathbf {\nabla }\cdot \mathbf{j} (\mathbf{r} ,t)=0$, $\omega _{n0}(\rho _\mathbf{q} )_{0n}=\mathbf{q} \cdot (\mathbf{j} _\mathbf{q })_{0n}$ and $\omega _{n0}(\rho _\mathbf{q} )_{n0}=-\mathbf{q} \cdot (\mathbf{j} _\mathbf{q })_{n0}$, we can obtain

19 $$\begin{aligned} \mathbf{q} \cdot \mathbf{B} (\mathbf{q} ,0)&=-\sum _n[\langle 0|\rho _\mathbf{q} |n\rangle \langle n|{\hat{\Delta }}^{\dag }_\mathbf{q }|0\rangle -\langle 0|{\hat{\Delta }}^{\dag }_\mathbf{q} |n\rangle \langle n|\rho _\mathbf{q} |0\rangle ],\nonumber \\&=-\langle 0|[\rho _\mathbf{q} ,{\hat{\Delta }}^{\dag }_\mathbf{q} ]|0\rangle =-2\langle 0|{\hat{\Delta }}^{\dag }_\mathbf{q =\mathbf{0} }|0\rangle =-2\Delta , \end{aligned}$$

where density fluctuation operator $\rho _\mathbf{q} =\sum _{\sigma \mathbf{k} } \psi ^{\dag }_{\sigma \mathbf{k} }\psi _{\sigma \mathbf{k} +\mathbf{q} }$ and we use the fact that $\langle {\hat{\Delta }}^{\dag }_\mathbf{q =0}\rangle =\Delta ^*=\Delta$. So

20 $$\begin{aligned}&\mathbf{q} \cdot \delta \mathbf{j} (r)=-2[\xi e^{i\mathbf{q} \cdot \mathbf{r} }\Delta +h.c.]. \end{aligned}$$

For isotropic system, further assuming $\mathbf{q} \parallel \mathbf{B} \propto \delta \mathbf{j}$ and using Eq. (14), so we get

21 $$\begin{aligned} \delta \mathbf{j }(\mathbf{r })&=-\frac{2\mathbf{q }\Delta }{q^2}\left[ \xi e^{i\mathbf{q }\cdot \mathbf{r }}+\xi ^* e^{-i\mathbf{q }\cdot \mathbf{r }}\right] ,\nonumber \\&=-2\Delta \frac{\mathbf{q }}{q^2} 2\alpha cos(\mathbf{q }\cdot \mathbf{r }+\phi )=-\frac{2\Delta }{q^2} \frac{\mathbf {\nabla }\delta \Delta (\mathbf{r })}{iG_{II}(\mathbf{q },0)}. \end{aligned}$$

Here we further use Eq. (5), and then get

22 $$\begin{aligned}&\delta \mathbf{j} (\mathbf{r} )= -\frac{4\Delta ^{2}}{q^2} \frac{\mathbf {\nabla }\delta \theta (\mathbf{r} )}{G_{II}(\mathbf{q} ,0)}=-\frac{4\Delta ^{2}m}{q^2} \frac{ \mathbf{v} _s}{G_{II}(\mathbf{q} ,0)}. \end{aligned}$$

Using Eq. (6), i.e., $\delta \mathbf{j} (\mathbf{r} )\equiv \rho _s \mathbf{v} _s$, the Josephson relation for conventional two-component Fermions is obtained

23 $$\begin{aligned} \rho _s=-\mathrm{lim}_{{\hbox {q}\rightarrow 0}}\frac{4\Delta ^{2}m}{q^2 G_{II}(\mathbf{q} ,0)} , \end{aligned}$$

which is consistent with the Taylor’s^11^ and Dawson et al.’s^12^ results.

#### General Josephson relation for multi-component fermions

For a multi-component (or multi-band) fermion superfluid, the order parameter $\Delta _{\alpha \beta }$ can be written as

24 $$\begin{aligned} \Delta _{\alpha \beta }=\langle {\hat{\Delta }}_{\alpha \beta }(\mathbf{r} )\rangle , \end{aligned}$$

where the operator of order parameter ${\hat{\Delta }}_{\alpha \beta }(\mathbf{r} )=g_{\alpha \beta }\psi _{\alpha }(\mathbf{r} )\psi _{\beta }(\mathbf{r} )$, $\psi _{\alpha (\beta )}$ is the field operator for $\alpha (\beta )$-th component, and $g_{\alpha \beta }$ is interaction parameter between $\alpha$-th and $\beta$-th components. The above equation indicates that the number of superfluid order parameter may be an arbitrary integer $m\ge 1$ ($m\in Z$) in a multi-component fermion superfluid. In such a case, we need a general perturbation Hamiltonian

25 $$\begin{aligned}&H^{\prime}=\int d^3r\{e^{i(\mathbf{q} \cdot \mathbf{r} -\omega t+\epsilon t)} {\hat{\Delta }}^{\dag }(\mathbf{r} )\cdot\xi +e^{-i(\mathbf{q} \cdot \mathbf{r} -\omega t+\epsilon t)}\xi ^{\dag }\cdot{\hat{\Delta }}(\mathbf{r} )\},\nonumber \\&\quad ={\hat{\Delta }}^{\dag }_\mathbf{q }\cdot\xi e^{-i\omega t+\epsilon t}+\xi ^{\dag }\cdot{\hat{\Delta }}_\mathbf{q }e^{i\omega t+\epsilon t}, \end{aligned}$$

where we relabel the operator of order parameter with ${\hat{\Delta }}_{i}(i=1,2,...,m)$ and introduce column vectors ${\hat{\Delta }}(\mathbf{r} )=\{{\hat{\Delta }}_1(\mathbf{r} ),{\hat{\Delta }}_2(\mathbf{r} ),...,{\hat{\Delta }}_m(\mathbf{r} ) \}^\mathbf{t}$, $\xi =\{ \xi _1,\xi _2,...,\xi _m \}^\mathbf{t}$ and $\{...\}^t$ denotes matrix transpose and dot $\cdot$ is the multiplication of matrices.

In the above subsection, the definition of superfluid velocity involves particle mass *m*. However, the superfluid system we considered here can be a general many-body system with complex energy spectra. For an arbitrarily general multi-component (or multi-band) system, it may be difficult to introduce notion of mass. Furthermore, the superfluid velocity may be unable to be defined unambiguously. In the following manuscript, the superfluid density is simply defined as the coefficient before the gradient of phase in current, i.e.,

26 $$\begin{aligned} \delta \mathbf{j} =\rho _s \mathbf {\nabla } \delta \theta (\mathbf{r} ). \end{aligned}$$

For a general multi-component superfluid system, the phase variation of order parameter $\delta \theta$ is well-defined. Consequently, the physical meaning of superfluid density $\rho _s$ is also clear. In fact, the above definition of superfluid density is also consistent with the phase twist method in lattice system^46^.

Similarly using perturbation theory and translational invariance, we can get the change of order parameter

27 $$\begin{aligned} \delta \Delta _{i}(\mathbf{r} )&=\sum _{j}[e^{i\mathbf{q} \cdot \mathbf{r} -i(\omega +i\epsilon )t} G_{IIij}(\mathbf{q} ,\omega +i\epsilon )\xi _{j}\nonumber \\&\quad +e^{-i\mathbf{q} \cdot \mathbf{r} +i(\omega -i\epsilon )t} F_{IIji}(\mathbf{q} ,\omega -i\epsilon ) \xi _{j}^{*}], \end{aligned}$$

where

28 $$\begin{aligned}&G_{IIij}(\mathbf{q} ,\omega +i\epsilon )\nonumber \\&\quad =\sum _n\left[ \frac{\langle 0|{\hat{\Delta }}_{i \mathbf{q} }|n\rangle \langle n|{\hat{\Delta }}^{\dag }_{j \mathbf{q} }|0\rangle }{\omega +i\epsilon -\omega _{n0}}-\frac{\langle 0|{\hat{\Delta }}^{\dag }_{j \mathbf{q} }|n\rangle \langle n|{\hat{\Delta }}_{i \mathbf{q} }|0\rangle }{\omega +i\epsilon +\omega _{n0}}\right] ,\nonumber \\&F_{IIji}(\mathbf{q} ,\omega -i\epsilon )\nonumber \\&\quad =\sum _n\left[ \frac{\langle 0|{\hat{\Delta }}_{j,\mathbf{q} }|n\rangle \langle n|{\hat{\Delta }}_{i,-\mathbf{q} }|0\rangle }{\omega -i\epsilon -\omega _{n0}}-\frac{\langle 0|{\hat{\Delta }}_{i,-\mathbf{q} }|n\rangle \langle n|{\hat{\Delta }}_{j,\mathbf{q} }|0\rangle }{\omega -i\epsilon +\omega _{n0}}\right] , \end{aligned}$$

are two-particle normal (anomalous) Green function matrix elements and index $i (j)=1,2,...,m$. $|n\rangle$ is also eigenstate of (canonical) momentum $\mathbf{P} =\sum _{k,\sigma }{} \mathbf{k} \psi ^{\dag }_{\sigma k}\psi _{\sigma k}$, i.e., $\mathbf{P} |n=\mathbf{q} _n|n\rangle$.

In a general multi-component fermion superfluid, the current fluctuation operator can be written as $\mathbf{j} _\mathbf{q }=\sum _{\alpha \beta \mathbf{k} } T_{\alpha \beta }(\mathbf{k} ,\mathbf{q} )\psi ^{\dag }_{\alpha \mathbf{k} }\psi _{\beta \mathbf{k} +\mathbf{q} }$, where $T_{\alpha \beta }(\mathbf{k} ,\mathbf{q} )$ is a function of $\mathbf{k}$ and $\mathbf{q}$. Its specific form is determined by the kinetic energy part of Hamiltonian. For usual parabola dispersion energy, e.g., as shown in Eq. (3), $T_{\alpha \beta }(\mathbf{k} ,\mathbf{q} )=\delta _{\alpha \beta }[\mathbf{k} +\mathbf{q} /2]/m$. Similarly as above section, one gets current

29 $$\begin{aligned} \delta \mathbf{j} (\mathbf{r} )=e^{i\mathbf{q} \cdot \mathbf{r} -i(\omega +i\epsilon )t} \mathbf{B} ^{t}(q,\omega +i\epsilon ).\xi +h.c., \end{aligned}$$

and here we introduce a $m\times 1$ column vector **B** and its *j*-th component

30 $$\begin{aligned}&\mathbf{B} _{j}(q,\omega +i\epsilon )\nonumber \\&\quad =\sum _n\left[ \frac{\langle 0|\mathbf{j} _\mathbf{q} |n\rangle \langle n|{\hat{\Delta }}_{j\mathbf{q} }^{\dag }|0\rangle }{\omega +i\epsilon -\omega _{n0}}-\frac{\langle 0|{\hat{\Delta }}_{j\mathbf{q} }^{\dag }|n\rangle \langle n|\mathbf{j} _\mathbf{q} |0\rangle }{\omega +i\epsilon +\omega _{n0}}\right] . \end{aligned}$$

When $$\omega \pm i\epsilon = 0$$,

31 $$\begin{aligned}&\delta \Delta (\mathbf{r} )=e^{i\mathbf{q} \cdot \mathbf{r} } G_{II}(\mathbf{q} ,0).\xi +e^{-i\mathbf{q} \cdot \mathbf{r} } F_{II}^\mathbf{t }(\mathbf{q} ,0).\xi ^{*},\nonumber \\&\delta \mathbf{j} (\mathbf{r} )=e^{i\mathbf{q} \cdot \mathbf{r} } \mathbf{B} ^t(\mathbf{q} ,0).\xi +h.c. \end{aligned}$$

Similarly using continuity equation $\frac{\partial \rho (\mathbf{r} ,t)}{\partial t}+\mathbf {\nabla }\cdot \mathbf{j} (\mathbf{r} ,t)=0$, $\omega _{n0}(\rho _\mathbf{q} )_{0n}=\mathbf{q} \cdot (\mathbf{j} _\mathbf{q })_{0n}$, $\omega _{n0}(\rho _\mathbf{q} )_{n0}=-\mathbf{q} \cdot (\mathbf{j} _\mathbf{q })_{n0}$, and assuming $\mathbf{q} \parallel \mathbf{B} \propto \delta \mathbf{j}$ for isotropic system, we get

32 $$\begin{aligned} \mathbf{B} ^{t}(\mathbf{q} ,0)=-\frac{2\mathbf{q} }{q^2}\{\Delta ^{*}_{1},\Delta ^{*}_{2},...,\Delta ^{*}_{m}\}. \end{aligned}$$

Here density fluctuation operator takes the same form as that in two-component case, i.e., $\rho _\mathbf{q} =\sum _{\sigma \mathbf{k} } \psi ^{\dag }_{\sigma \mathbf{k} }\psi _{\sigma \mathbf{k} +\mathbf{q} }$.

Introducing $x(\mathbf{r} )=e^{i\mathbf{q} \cdot \mathbf{r} }\{\xi _{1},\xi _{2},...,\xi _{m} \}^\mathbf{t}$, $\delta \Delta (\mathbf{r} )=\{\delta \Delta _{1}(\mathbf{r} ), \delta \Delta _{2}(\mathbf{r} ),...,\delta \Delta _{m}(\mathbf{r} )\}^\mathbf{t}$, the above Eq. (31) can be written as

33 $$\begin{aligned}&\left( \!\!\! \begin{array}{cccc} \delta \Delta (\mathbf{r} ) \\ \delta \Delta (\mathbf{r} )^{*} \\ \end{array}\!\!\!\right) \!\!=\!\!\left( \!\!\! \begin{array}{cccc} G_{II}(\mathbf{q} ,0) &{} F_{II}^\mathbf{t }(\mathbf{q} ,0) \\ F_{II}^\mathbf{t *}(\mathbf{q} ,0)&{} G_{II}^{*}(\mathbf{q} ,0)\\ \end{array}\!\!\!\right) _{2m\times 2m}.\left( \!\!\! \begin{array}{cccc} x \\ x^* \\ \end{array}\!\!\!\right) \nonumber \\&\equiv \mathbf{G} _\mathbf{II }(\mathbf{q} ).\left( \!\!\! \begin{array}{cccc} x \\ x^* \\ \end{array}\!\!\!\right) =2i\delta \theta \left( \!\!\! \begin{array}{cccc} \Delta \\ -\Delta ^* \\ \end{array}\!\!\!\right) \end{aligned}$$

and

34 $$\begin{aligned}&\delta \mathbf{j} (\mathbf{r} )\!\!=\!\!-\frac{2\mathbf{q} }{q^2}\left( \begin{array}{cccc} \Delta ^{t*} &{}\Delta ^{t} \\ \end{array}\right) .\!\!\left( \!\!\! \begin{array}{cccc} (I)_{m\times m} &{} 0_{m\times m} \\ 0_{m\times m} &{} (I)_{m\times m}\\ \end{array}\!\!\!\right) .\left( \begin{array}{cccc} x \\ x^* \\ \end{array}\!\!\!\right) , \end{aligned}$$

where $(I)_{m\times m}$ is a $m\times m$ identity matrix and we define coefficient matrix

35 $$\begin{aligned} \mathbf{G} _\mathbf{II }(\mathbf{q} )\equiv \!\!\left( \!\!\! \begin{array}{cccc} G_{II}(\mathbf{q} ,0) &{} F_{II}^\mathbf{t} (\mathbf{q} ,0) \\ F_{II}^{t*}(\mathbf{q} ,0)&{} G_{II}^{*}(\mathbf{q} ,0)\\ \end{array}\!\!\!\right) _{2m\times 2m} , \end{aligned}$$

which is a $2m\times 2m$ matrix and one should not confuse with normal Green function $G_{II}(\mathbf{q} ,0)$, which is a $m\times m$ matrix. If $\mathbf{G} _\mathbf{II }$ has inverse (determinant $Det|\mathbf{G} _\mathbf{II }|\ne 0$), using $\mathbf{q} x(\mathbf{r} )=-i \mathbf {\nabla } x$, $\mathbf{q} x^*(\mathbf{r} )=i \mathbf {\nabla } x^*$ and Eqs. (33) and (34), we get

36 $$\begin{aligned}&\delta \mathbf{j} (\mathbf{r} )=-\frac{4\mathbf {\nabla }\delta \theta (\mathbf{r} )}{q^2}(\Delta ^{t*},\Delta ^{t})\nonumber \\&.\!\!\left( \!\!\! \begin{array}{cccc} I &{} 0 \\ 0&{} -I\\ \end{array}\!\!\!\right) .\mathbf{G} _\mathbf{II }^{-1}.\!\!\left( \!\!\! \begin{array}{cccc} I &{} 0 \\ 0&{} -I\\ \end{array}\!\!\!\right) .\left( \begin{array}{cccc} \Delta \\ \Delta ^*\\ \end{array}\right) . \end{aligned}$$

Using Eq. (26), i.e., $\delta \mathbf{j} (\mathbf{r} )\equiv \rho _s \mathbf {\nabla } \delta \theta$, we get a general Josephson relation for fermion superfluid

37 $$\begin{aligned} \rho _s=\mathrm{lim}_{{\hbox {q}\rightarrow 0}}\frac{-4}{q^2}(\Delta ^{t*},\Delta ^t).\!\!\left( \!\!\! \begin{array}{cccc} I &{} 0 \\ 0&{} -I\\ \end{array}\!\!\!\right) .\mathbf{G} _\mathbf{II }^{-1}(\mathbf{q} ).\!\!\left( \!\!\! \begin{array}{cccc} I &{} 0 \\ 0&{} -I\\ \end{array}\!\!\!\right) \!\!.\!\!\left( \!\! \begin{array}{cccc} \Delta \\ \Delta ^*\\ \end{array}\!\!\right) . \end{aligned}$$

The Eq. (37) gives a way to calculate the superfluid density in terms of two-particle Green functions, which is also the main result in this work. It should be emphasized that the above result Eq. (37) only relies on the definition of superfluid density Eq. (26), the existence of superfluid order parameter ($\Delta \ne 0$), translational invariance and continuity equation for particle number.

In addition, even though Eq. (37) is obtained in a translational invariant system (the momentum is a good quantum number), the above formula can be applied equally to lattice system as long as the superfluid state has lattice translation symmetry (see also “Summary” section). This is because, we know for lattice system, the engenstates can be classified by lattice momentum. In the above derivation, on the one hand, we need to identify the (canonical) momentum $\mathbf{P} =\sum _{k,\sigma }{} \mathbf{k} \psi ^{\dag }_{\sigma k}\psi _{\sigma k}$ with the lattice momentum. On the other hand, the superfluid density may show some anisotropy in lattice system. We can use a similar method as done in bosonic system^39^ to deal with the anisotropy in lattice. In such a case, the superfluid density is usually a rank-two tensor and depends on direction of $\mathbf{q}$.

In isotropic case, the superfluid density is a scalar, i.e., $\rho _s=\mathbf{diag} \{\rho _s ,\rho _s ,\rho _s \}$ which does not depend on direction of $\mathbf{q}$. For generally anisotropic system (for example, Lieb lattice system^36^, the spin-orbital coupled cold atoms^47^, etc), the induced current $\delta \mathbf{j}$ can be expressed in terms of vector $\mathbf{q}$ and a rank-two tensor *m* (generally $\delta \mathbf{j}$ is not parallel to $\mathbf{q}$ any more), namely

38 $$\begin{aligned} \delta \mathbf{j} _{i}(\mathbf{r} )=\sum _{j=x,y,z} m_{ij}{} \mathbf{q} _j, \end{aligned}$$

where indices $i(j)=x,y,z$ in three dimensional space. In order to get Josephson relation in anisotropic system, we introduce the superfluid density $\rho _{s}({\hat{q}})$ along ${\hat{q}}$ direction, i.e., ${\hat{q}}\cdot \delta \mathbf{j} (\mathbf{r} )\equiv \rho _{s}({\hat{q}}) ({\hat{q}}\cdot \mathbf {\nabla } \delta \theta )$, where ${\hat{q}}\equiv \mathbf{q} /q$ is unit vector along $\mathbf{q}$ direction. From Eqs. (31) and (38), we get

$$\begin{aligned} {\hat{q}}\cdot \delta \mathbf{j }(\mathbf{r })&=\sum _{ij=x,y,z}qm_{ij}{\hat{q}}_i {\hat{q}}_j\\&=-\frac{2q}{q^2}\left( \begin{array}{cccc} \Delta ^{t*} &{}\Delta ^{t} \\ \end{array}\right) .\!\!\left( \!\!\! \begin{array}{cccc} (I)_{m\times m} &{} 0_{m\times m} \\ 0_{m\times m} &{} (I)_{m\times m}\\ \end{array}\!\!\!\right) .\left( \begin{array}{cccc} x \\ x^* \\ \end{array}\!\!\!\right) , \end{aligned}$$

which is exactly similar to Eq. (34) of isotropic case. Taking $q x(\mathbf{r} )=-i {\hat{q}}\cdot \mathbf {\nabla } x$, $q x^*(\mathbf{r} )=i {\hat{q}}\cdot \mathbf {\nabla } x^*$ and Eq. (33) into account, the following discussions for anisotropic case are same with that in the isotropic case. So the superfluid density along direction ${\hat{q}}$ is

39 $$\begin{aligned} \rho _s({\hat{q}})=\mathrm{lim}_{{\hbox {q}\rightarrow 0}}\frac{-4}{q^2}(\Delta ^{t*},\Delta ^t).\!\!\left( \!\!\! \begin{array}{cccc} I &{} 0 \\ 0&{} -I\\ \end{array}\!\!\!\right) .\mathbf{G} _\mathbf{II }^{-1}(\mathbf{q} ).\!\!\left( \!\!\! \begin{array}{cccc} I &{} 0 \\ 0&{} -I\\ \end{array}\!\!\!\right) \!\!.\!\!\left( \!\! \begin{array}{cccc} \Delta \\ \Delta ^*\\ \end{array}\!\!\right) , \end{aligned}$$

which usually depends on the direction of ${\hat{q}}$. Furthermore, from the superfluid density along an arbitrary direction ${\hat{q}}$, i.e.,

$$\begin{aligned} \rho _s({\hat{q}})=\sum _{i,j=x,y,z}\rho _{s;ij} {\hat{q}}_i {\hat{q}}_j, \end{aligned}$$

it is not difficult to construct a rank-two superfluid density tensor $\rho _{s;ij}$.

### Discussions

In the above derivations, we get the superfluid density in terms of two-particle Green function [the Josephson relation Eq. (37)]. In this section, we will show that for some cases, the above equation can be further simplified. To be specific, if the system has only one gapless collective mode, the superfluid density can be give by the trace of two-particle Green function. When the inversion symmetry is present, the superfluid density can be expressed in terms of the fluctuation matrix of superfluid order parameters.

#### Only one gapless collective mode

When a system has unique gapless excitation near ground state, e.g., phonon, the Josephson relation can be generalized to a multi-component system with a phase operator method as done in bosonic case^39^. Here we know near the ground state, the phonon corresponds to total density oscillation. Due to the presence of superfluid order parameter, the density oscillation would couple phase oscillation of order parameter^48^. Furthermore, all the superfluid order parameters should share a common phase variation, i.e., $\delta \theta _i(\mathbf{r} )=\delta \theta (\mathbf{r} )$. On the other hand, near the ground state, similarly as Eq. (5), the fluctuation operator of superfluid order parameters may be expressed in terms of phase operator ${\hat{\theta }}$^40^

40 $$\begin{aligned} \delta {\hat{\Delta }}_{i}(\mathbf{r} )\simeq 2i\Delta _i\delta {\hat{\theta }}(\mathbf{r} ), \end{aligned}$$

where $\Delta _{i}$ is the *i*-th superfluid order parameter in ground state. Under perturbation $H'$ [see Eq. (25)], the variations of order parameters can be obtained by averaging Eq. (40) with respect to the perturbed ground state. Consequently, the variations of order parameters are $\delta \Delta _{i}=2i\Delta _{i}\delta \theta (\mathbf{r} )$ with $\delta \theta (\mathbf{r} )=\langle \delta \hat{ \theta }(\mathbf{r} )\rangle$.

From the above equation, we get the fluctuation operators of order parameters in momentum space

$$\begin{aligned}&{\hat{\Delta }}_{i,\mathbf{q} }=2i\Delta _i{\hat{\theta }}_\mathbf{q },\\&{\hat{\Delta }}^{\dag }_{i,\mathbf{q} }=-2i\Delta ^{*}_{i}{\hat{\theta }}^{\dag }_\mathbf{q }=-2i\Delta ^{*}_{i}{\hat{\theta }}_{-\mathbf{q} }.\\&{\hat{\Delta }}_{i,-\mathbf{q} }=2i\Delta _i{\hat{\theta }}_{-\mathbf{q} },\\&{\hat{\Delta }}^{\dag }_{i,-\mathbf{q} }=-2i\Delta ^{*}_{i}{\hat{\theta }}^{\dag }_{-\mathbf{q} }=-2i\Delta ^{*}_{i}{\hat{\theta }}_\mathbf{q }, \end{aligned}$$

where we use ${\hat{\theta }}^{\dag }_\mathbf{q }={\hat{\theta }}_{-\mathbf{q} }$ for real phase field $\theta (\mathbf{r} )$ ($\mathbf{q} \ne 0$) . From definitions of the $G_{II}$ and $F_{II}$ in Eq. (28), we get

41 $$\begin{aligned}&G_{IIij}(\mathbf{q} ,0)=-4Z\Delta _i\Delta ^{*}_{j},\nonumber \\&F_{IIji}(\mathbf{q} ,0)=4Z\Delta _{j}\Delta _{i} , \end{aligned}$$

where $Z\equiv \sum _n[\frac{|\langle 0|{\hat{\theta }}_\mathbf{q }|n\rangle |^2}{\omega _{n0}}+\frac{|\langle 0|{\hat{\theta }}_{-\mathbf{q} }|n\rangle |^2}{\omega _{n0}}]>0$ is a real number. When the number of superfluid order parameters is one and $\Delta$ is real, the relation $F_{II}(\mathbf{q} ,0)=-G_{II}(\mathbf{q} ,0)$ holds ($q\rightarrow 0$) in conventional two-component fermion superfluid.

From Eqs. (33) and (34), we get

$$\begin{aligned} 2i\delta \theta (\mathbf{r })&=-4Z\sum _i [ \Delta ^{*}_{i} x_i-\Delta _i x^{*}_{i}],\\&=-4Z\sum _i 2i\alpha _i sin(\mathbf{q }\cdot \mathbf{r }+\phi _i),\\ \delta \mathbf{j }(\mathbf{r })&=-\frac{2\mathbf{q }}{q^2}\sum _{i}[ \Delta ^{*}_{i} x_i+\Delta _i x^{*}_{i}],\\&=-\frac{2\mathbf{q }}{q^2}\sum _{i}[2\alpha _i cos(\mathbf{q }\cdot \mathbf{r }+\phi _i)]\\&=\frac{\mathbf {\nabla } \delta \theta (\mathbf{r })}{q^2Z}=\frac{\mathbf{v }_s}{q^2Z}, \end{aligned}$$

where we set $\Delta ^{*}_{i} \xi _i\equiv \alpha _i e^{i\phi _i}$ with amplitude $\alpha _{i}$, phase $\phi _{i}$. So the superfluid density is

42 $$\begin{aligned} \rho _s=\frac{1}{q^2Z}. \end{aligned}$$

On the other hand, we know

43 $$\begin{aligned} tr G_{II}(\mathbf{q} ,0)\equiv \sum ^{m}_{i=1} G_{II,ii}(\mathbf{q} ,0)=-4Z \sum ^{m}_{i=1}|\Delta _{i}|^2. \end{aligned}$$

So finally we get the Josephson relation

44 $$\begin{aligned} \rho _s=-\mathrm{lim}_{{\hbox {q}\rightarrow 0}}\frac{4\sum ^{m}_{i=1}|\Delta _{i}|^2}{q^2 trG_{II}(\mathbf{q} ,0)}, \end{aligned}$$

where $G_{II}(\mathbf{q} ,0)$ is two-particle normal Green function (matrix) at zero-frequency. It is found that the above Eq. (44) can be applied to the case of three superfluid parameters in dice lattice, where there exist one gapless phonon, and two gapped Leggett modes arising from the relative phase oscillations between different superfluid order parameters^21^. When the number of order parameters $m=1$, the above equation is reduced to the Josephson relation for conventional two component fermion superfluid^11,12^.

#### Superfluid density and pairing fluctuations

In this section, we would give a connection between the two-particle Green function and the pairing fluctuation matrix based on BCS mean field theory and Gaussian fluctuation approximation^11^. Furthermore, if the system has spatial inversion symmetry, the coefficient matrix $\mathbf{G} _\mathbf{II }$ is directly proportional to the inverse of pairing fluctuation matrix.

Fist of all, we discuss the results for conventional two-component fermion superfluid. In the following, we assume the pairing gap can be decomposed as the mean field value $\Delta$ and small fluctuation $\delta \Delta$ ($\Delta (\mathbf{r} )=\Delta +\delta \Delta$). Expanding action *S* to second order of $\delta \Delta$ , the partition function^49–51^

45 $$\begin{aligned}&Z\approx e^{-S_0}\int D \eta ^{\dag }_q D\eta _q e^{-\delta S}, \end{aligned}$$

where $S_0$ is the mean-field contribution and Gaussian fluctuation part

46 $$\begin{aligned} \delta S&=\frac{1}{2}\sum _\mathbf{q ,n}\eta ^{\dag }_q M(q)\eta _q,\nonumber \\&=\sum _\mathbf{q , n>0}\eta ^{\dag }_q\left( \begin{array}{cccc} M^o_{11}(q) &{} M^o_{12}(q) \\ M^o_{21}(q) &{} M^o_{22}(q) \end{array}\right) \eta _q, \end{aligned}$$

where pairing fluctuation field $\eta ^{\dag }_q=[\Delta ^{*}_q,\Delta _{-q}]$ and $q=(\mathbf{q} ,i\omega _n)$. $\omega _n=2n\pi /\beta$ ($n\in Z$) is Matsubara frequency, $\beta =1/T$ is inverse temperature. The fluctuation matrix *M* is a $2\times 2$ matrix

47 $$\begin{aligned}&M_{11}(\mathbf{q} ,i\omega _n)=\frac{1}{\beta }\sum _\mathbf{k ,n^{\prime}}G_{11}(k+q)G_{22}(k)+\frac{1}{g},\nonumber \\&M_{12}(\mathbf{q} ,i\omega _n)=\frac{1}{\beta}\sum _\mathbf{k ,n^{\prime}}G_{12}(k+q)G_{12}(k),\nonumber \\&M_{21}(\mathbf{q} ,i\omega _n)=M_{12}(\mathbf{q} ,i\omega _n),\nonumber \\&M_{22}(\mathbf{q} ,i\omega _n)=M_{11}(-\mathbf{q} ,-i\omega _n), \end{aligned}$$

where $G_{ij}(k)$ is matrix element of Nambu–Gorkov Green function. The collective modes are given by zeros of determinant $Det|M(\mathbf{q} ,i\omega _n\rightarrow \omega +i0^+)|=0$. As $q\rightarrow 0$, the collective mode is the Anderson–Bogoliubov phonon, which characterizes the density oscillations of superfluid. With the action $\delta S$ (Gaussian weight), the correlation function (average values of quadratic terms) can be calculated^52^, i.e.,

48 $$\begin{aligned}&\langle \Delta ^{*}_q \Delta _{q}\rangle =(M^{-1})_{11},\nonumber \\&\langle \Delta _{-q} \Delta _q \rangle =(M^{-1})_{12},\nonumber \\&\langle \Delta ^{*}_{q}\Delta ^{*}_{-q} \rangle =(M^{-1})_{21},\nonumber \\&\langle \Delta _{-q}\Delta ^{*}_{-q}\rangle =(M^{-1})_{22}. \end{aligned}$$

On the other hand, we know that the above correlation function has one extra minus sign in comparison with Green function. So the two-particle Green function can be obtained by

49 $$\begin{aligned}&-G_{II}(q)=\langle \Delta ^{*}_q \Delta _{q}\rangle =(M^{-1})_{11},\nonumber \\&-F_{II}(q)=\langle \Delta _{-q} \Delta _q \rangle =(M^{-1})_{12},\nonumber \\&-F^{*}_{II}(q)=\langle \Delta ^{*}_{q}\Delta ^{*}_{-q} \rangle =(M^{-1})_{21},\nonumber \\&-G_{II}(-q)=\langle \Delta _{-q}\Delta ^{*}_{-q}\rangle =(M^{-1})_{22}. \end{aligned}$$

In addition, if the system has inversion symmetry, i.e.,

50 $$\begin{aligned}&G_{II}(-\mathbf{q} ,i\omega _n)=G_{II}(\mathbf{q} ,i\omega _n),\nonumber \\&F_{II}(-\mathbf{q} ,i\omega _n)=F_{II}(\mathbf{q} ,\omega _n). \end{aligned}$$

Furthermore, according to Eq. (28), when $\omega \pm i\epsilon =0$, the Green function satisfy

51 $$\begin{aligned}&G^{*}_{II\sigma \sigma '}(\mathbf{q} ,0)=G_{II\sigma '\sigma }(\mathbf{q} ,0),\nonumber \\&F_{II\sigma \sigma '}(\mathbf{q} ,0)= F_{II\sigma '\sigma }(-\mathbf{q} ,0), \end{aligned}$$

then the coefficient matrix of Green function $\mathbf{G} _\mathbf{II }$ can be expressed in terms of the inverse of *M*, i.e.,

52 $$\begin{aligned}&\mathbf{G} _\mathbf{II }=\left( \!\!\! \begin{array}{cccc} G_{II}(q) &{} F_{II}(q) \\ F^{*}_{II}(q)&{} G^{*}_{II}(q)\\ \end{array}\!\!\!\right) ,\nonumber \\&=\left( \!\!\! \begin{array}{cccc} G_{II}(q) &{} F_{II}(q) \\ F^{*}_{II}(q)&{} G_{II}(-q)\\ \end{array}\!\!\!\right) =-M^{-1}. \end{aligned}$$

So the superfluid density

53 $$\begin{aligned} \rho _s&=\mathrm{lim}_{{\hbox {q}\rightarrow 0}}\frac{-4}{q^2}(\Delta ^{t*},\Delta ^t).\!\!\left( \!\!\! \begin{array}{cccc} I &{} 0 \\ 0&{} -I\\ \end{array}\!\!\!\right) .\mathbf{G} _\mathbf{II }^{-1}.\!\!\left( \!\!\! \begin{array}{cccc} I &{} 0 \\ 0&{} -I\\ \end{array}\!\!\!\right) \!\!.\!\!\left( \!\! \begin{array}{cccc} \Delta \\ \Delta ^*\\ \end{array}\!\!\right) ,\nonumber \\&=\mathrm{lim}_{{\hbox {q}\rightarrow 0}}\frac{4}{q^2}(\Delta ^{t*},\Delta ^t).\!\!\left( \!\!\! \begin{array}{cccc} I &{} 0 \\ 0&{} -I\\ \end{array}\!\!\!\right) .M(q).\!\!\left( \!\!\! \begin{array}{cccc} I &{} 0 \\ 0&{} -I\\ \end{array}\!\!\!\right) \!\!.\!\!\left( \!\! \begin{array}{cccc} \Delta \\ \Delta ^*\\ \end{array}\!\!\right) . \end{aligned}$$

Next, for the case of several superfluid order parameters, the proof is similar. Assuming the number of superfluid order parameters is an arbitrary integer *m*, then Gaussian fluctuation part of action

54 $$\begin{aligned} \delta S=\sum _\mathbf{q ,n>0}\eta ^{\dag }_q M(q)\eta _q, \end{aligned}$$

where *M*(*q*) is a $2m\times 2m$ fluctuation matrix, and pairing fluctuation field $\eta ^{\dag }_q=[\Delta ^{*}_{1q},\Delta ^{*}_{2q},...,\Delta ^{*}_{mq},\Delta _{1,-q},\Delta _{2,-q},...,\Delta _{m,-q}]$ . With the action $\delta S$ (Gaussian weight), the correlation functions (and the matrix elements of Green function) can be calculated, i.e.,

55 $$\begin{aligned}&-G_{II,ij}(q)=\langle \Delta ^{*}_{jq} \Delta _{iq}\rangle =(M^{-1})_{i,j},\nonumber \\&-F_{II,ij}(q)=\langle \Delta _{j,-q} \Delta _{iq} \rangle =(M^{-1})_{i,m+j},\nonumber \\&-F^{*}_{II,ji}(q)=\langle \Delta ^{*}_{jq}\Delta ^{*}_{i,-q} \rangle =(M^{-1})_{m+i,m+j},\nonumber \\&-G_{II,ji}(-q)=\langle \Delta _{j,-q}\Delta ^{*}_{i,-q}\rangle =(M^{-1})_{m+i,m+j}. \end{aligned}$$

Furthermore, in the presence of inverse symmetry, the coefficient matrix can be obtained in terms of the fluctuation matrix, i.e., $\mathbf{G} _\mathbf{II }=-M^{-1}$. So the superfluid density is given by

56 $$\begin{aligned} \rho _s = {lim}_{{\hbox {q}\rightarrow 0}}\frac{4}{q^2}(\Delta ^{t*},\Delta ^t).\!\!\left( \!\!\! \begin{array}{cccc} I &{} 0 \\ 0&{} -I\\ \end{array}\!\!\!\right) .M(q).\!\!\left( \!\!\! \begin{array}{cccc} I &{} 0 \\ 0&{} -I\\ \end{array}\!\!\!\right) \!\!.\!\!\left( \!\! \begin{array}{cccc} \Delta \\ \Delta ^*\\ \end{array}\!\!\right) . \end{aligned}$$

During the derivations of Eqs. (37), (44) and (56), we can see that the superfluid density is mainly related to the low energy collective excitations (e.g., phonon for neutral fermion superfluid) and the behaviors of Green functions at long wave length limit. In addition, it is found that the superfluid density in a multi-band fermion superfluid can be divided into two parts, one is the conventional part, which arises from the diagonal matrix elements of current operator, and the other one is the off-diagonal terms (the so called geometric part^36,46^), which connects with the geometric metric tensor of Bloch states^53^. The geometric part plays an important role in the understanding of superfluidity of flat band, where the conventional part is negligible^36^. In addition, it is expected that some other physical quantities, for example, sound velocity^20^ should also have similar geometric part in a multi-component (or multi-band) superfluid. The Josephson relations [Eqs. (37), (44) and (56)] give the total superfluid density, which not only includes conventional part, but also the geometric part of a multi-band fermion superfluid.

The non-vanishing superfluid density in superconductor can result in the perfect diamagnetism (the Meissner effect)^54^. The penetration depth of a magnetic field ($\lambda$) is related to the superfluid density through relation $\rho _s\propto 1/\lambda ^2$. So the superfluid density can be experimentally obtained by measuring the penetration depth of magnetic field in superconductor. On the hand, for two-dimensional high-$T_c$ superconductors, the superfluid density at zero-temperature is related to the superfluid transition temperature by $T_c\propto \rho _s(T=0)$ (the so called Uemura relation^55^ . So the superfluid density can be also evaluated by measuring the superconductor transition temperature $T_c$. For neutral superfluid system, the superfluid density would result in a reduction of moment of inertia of system (from its classical value). So the superfluid density can be obtained through measuring the moment of inertia of superfluid system^56,57^. In order to measure the moment of inertia of atomic gas, an optical method is proposed through imparting non-zero angular momentum into cold atom gas^58^.

### An example: superfluid density in Haldane–Hubbard model

As an application of the Josephon relations, e.g., Eqs. (44) and (56), the superfluid density is calculated for Haldane–Hubbard model in two-component Fermi gas with on-site attractive interaction $-U$ ($U>0$)^59^. The Hamiltonian for Haldane-Hubbard model is

57 $$\begin{aligned} H=\sum _{ij\sigma }t_{ij}c_{i}^{\dag }c_j+\sum _{i\sigma }(M\epsilon _i-\mu )n_{i\sigma }-U\sum _in_{i\uparrow }n_{i\downarrow }, \end{aligned}$$

where $\mu$ is chemical potential, and $n_{i\sigma }=c^{\dag }_{i\sigma }c_{i\sigma }$ is particle number operator. $\epsilon _i=1(-1)$ for sublattice A (B) and *M* is energy offset between sublattice A and B. $t_{ij}$ is hopping amplitude between lattice sites *i* and *j*, which is *t* for nearest neighbor sites, $t'e^{-i\phi }(t'e^{i\phi })$ for clockwise (anticlockwise) hopping between next-nearest neighbor sites^59^. The distance between nearest neighbor sites is *a*.

In the following, we focus on the case of $\phi =\pi /2$ and $M=0$, where the inversion symmetry is not broken. Due to the presence of two sublattices, Haldane model is a two-band fermion system. In addition, there exist two superfluid order parameters for two sublattices, i.g.,

$$\begin{aligned}&\Delta _{i,A}=\Delta _{i,A\downarrow ;i,A\uparrow }=-U\langle c_{i,A\downarrow }c_{i,A\uparrow }\rangle ,\\&\Delta _{i,B}=\Delta _{i,B\downarrow ;i,B\uparrow }=-U\langle c_{i,B\downarrow }c_{i,B\uparrow }\rangle , \end{aligned}$$

where *i* is unit cell index, $\downarrow (\uparrow )$ are two spin component indices. Furthermore, when $M=0$, the two order parameters are equal, e.g., $\Delta _{i,A}=\Delta _{i,B}=\Delta$. When the filling factor $n=2$ (2 particles per unit cell in half-filling), for weakly interacting case, the system is a band insulator, and the superfluid order parameter vanishes ($\Delta =0$). Only when the interaction is strong enough, the system enters a superfluid phase ($\Delta \ne 0$). Away from the half-filling, the system is usually in a superfluid phase^59^. In addition, it is found that, there exists only one gapless excitation near $q=0$, which corresponds to total density oscillations. The superfluid density can be also calculated with current–current correlation functions^9,34,47^ or phase twist method^46,60^. Assuming the superfluid order parameters undergo a phase variation, e.g, $\triangle _{iA(B)}\rightarrow \Delta _{iA(B)}e^{2i\mathbf{q} \cdot \mathbf{r} _{iA(B)}}$, the superfluid density (particle number per unit cell) tensor $\rho _{sij}$ can be written as

58 $$\begin{aligned} \rho _{sij}= \frac{\partial ^2 \Omega (\mathbf{q} )}{\partial q_i \partial q_j}|_{q\rightarrow 0}, \end{aligned}$$

where $\Omega$ is thermodynamical potential (per unit cell) in grand canonical ensemble.

**Figure 1:**
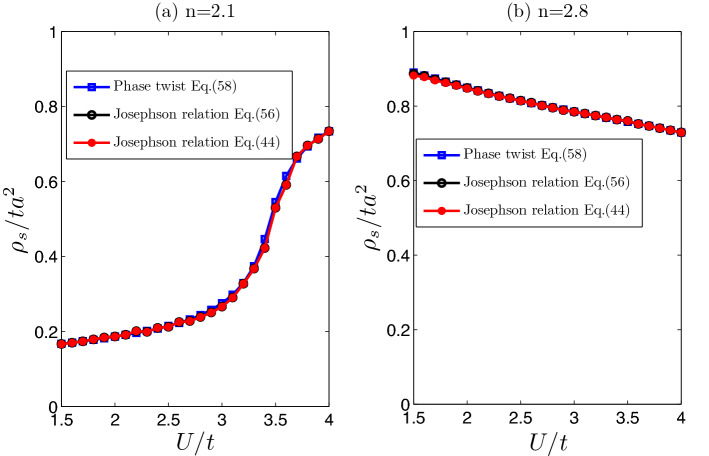
Superfluid densities (particle number per unit cell) are plotted as functions of interaction *U*. (**a**) Filling factor $n=2.1$, $t'=0.15t$, $M=0$ and $\phi =\pi /2$; (**b**) filling factor $n=2.8$, $t'=0.15t$, $M=0$ and $\phi =\pi /2$. The the superfluid density in the three curves are obtained through three different formulas, i.e., Eqs. (58), (56) and (44).

**Figure 2:**
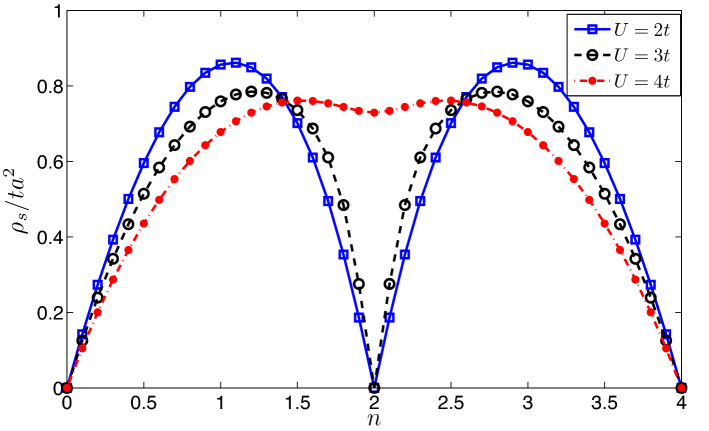
Superfluid densities (particle number per unit cell) are plotted as functions of the filling factor *n* (particle number per unit cell) with $t'=0.15t$, $M=0$ and $\phi =\pi /2$. The three curves correspond to interaction $U=2t,3t$ and 4t, respectively.

Figure 1 shows the evolutions of superfluid density as the interaction increases. The superfluid density is obtained with three different formulas, i.e. Eqs. (58), (56) and (44). First of all, it is found that the superfluid density is isotropic and behaves as a scalar in two-dimensional space, i.e., $\rho _{sij}=diag(\rho _s,\rho _s)$, which is a consequence of $C_3$ rotational symmetry of honeycomb lattice^46^. Secondly, the results from the Josephson relations [Eqs. (56), (44)] are consistent with the results from phase twist method [Eq. (58)]. In addition, When filling factor $n=2.1$, the superfluid density increases as the interaction gets strong. However when the filling factor $n=2.8$, the superfluid density gets smaller and smaller when the interaction increases. Such an interesting feature is also reflected in Fig. 2, that when the filling factor is near half-filling ($n=2$), the superfluid density increases as the interaction gets strong. However, when the filling is far away from half-filling, the situation is reversed (see Fig. 2).

Figure 2 shows the superfluid density as functions of filling factor ($n=0\rightarrow 4$). From the Fig. 2, we can see that the superfluid density is symmetric with respect to half-filling ($n=2$) due to particle-hole symmetry^59^. For weak interaction cases ($U=2t$ and $U= 3t$) and half filling, the system is a insulator (see Ref.^59^), the superfluid density vanishes (see Fig. 2). For fully occupied case ($n=4$ particles per unit cell), the system is equivalent to the completely empty case ($n=0$) due to the particle-hole symmetry, the system is also a insulator. Consequently, superfluid density is also zero. When the filling factor falls into the middle of upper band ($n\approx 3$), the superfluid density reaches its maximum. The appearance of double dome structure for weak interactions in Fig. 2 is in qualitative agreement with the results obtained through dynamical mean-field theory (DMFT) in Ref.^46^.

## Summary

In conclusion, we investigate the Josephson relation for a general multi-component fermion superfluid. It is found that the superfluid density is given in terms of two-particle Green functions. When the superfluid has only one gapless collective excitation, the Josephson relation can be simplified, which is given in terms of superfluid order parameters and trace of Green function. Within BCS mean field theory and Gaussian fluctuation approximation, the matrix elements of Green function can be given in terms of pairing fluctuation matrix elements. Furthermore, in the presence of inversion symmetry, it is shown that the two-particle Green function is directly proportional the inverse of pairing fluctuation matrix. The formulas for superfluid density are quite universal for generic multi-component fermion superfluids, which can be also applied to a multi-band superfluid with complex energy spectra in lattice.

Josephson relation for multi-component fermion superfluid provides a general method for calculations on superfluid densities in terms of two-particle Green functions and fluctuation matrix. Our work would be useful for investigations on the superfluid properties of multi-component (or multi-band) superfluid system with complex pairing structures.
